# HORDB a comprehensive database of peptide hormones

**DOI:** 10.1038/s41597-022-01287-5

**Published:** 2022-04-25

**Authors:** Ning Zhu, Fanyi Dong, Guobang Shi, Xingzhen Lao, Heng Zheng

**Affiliations:** School of Life Science and Technology, China Pharmaceutical University, 24 Tongjiaxiang, Nanjing, 210009 P. R. China

**Keywords:** Peptide hormones

## Abstract

Peptide hormones (also known as hormone peptides and polypeptide hormones) are hormones composed of peptides and are signal transduction molecules produced by a class of multicellular organisms. It plays an important role in the physiological and behavioral regulation of animals and humans as well as in the growth of plants. In order to promote the research on peptide hormones, we constructed HORDB database. The database currently has a total of 6024 entries, including 5729 peptide hormones, 40 peptide drugs and 255 marketed pharmaceutical preparations information. Each entry provided comprehensive information related to the peptide, including general information, sequence, activity, structure, physical information and literature information. We also added information on IC_50_, EC_50_, ED_50_, target, and whether or not the blood-brain barrier was crossed to the activity information note. In addition, HORDB integrates search and sequence analysis to facilitate user browsing and data analysis. We believe that the peptide hormones information collected by HORDB will promote the design and discovery of peptide hormones, All data are hosted and available in figshare 10.6084/m9.figshare.c.5522241.

## Abstract (summary)

**Table:** 

Measurement(s)	peptide hormone
Technology Type(s)	Comprehensive data website service
Factor Type(s)	peptide hormone

## Background & Summary

Since the appearance of insulin therapy in the 1920s, peptide have played an important role in the medical field^1^. One advantage of peptide drugs is that they are highly specific, have low levels of toxicity and provide a variety of drug targets^2,3^. Nowadays, more than 60 peptide drugs have been approved for marketing, and over 150 peptides enter the clinical stage^1^.

Peptide hormones as therapeutic drugs have been widely used in the medical field. For example, exenatide is a synthetic glucagon like peptide-1 (GLP-1) analogue exendin-4, which consists of 30 amino acids^4,5^. Studies have shown that exenatide is superior to metformin in reducing weight, controlling blood sugar, improving liver enzymes and weakening NAFLD in T2DM patients with NAFLD^6,7^. Thymosin α 1 is a peptide composed of 28 amino acids, which has the functions of improving cellular immune function and regulating various immune functions. ZADAXIN, a drug developed by thymosin alpha 1, is approved in 35 countries for the treatment of viral infections, immunodeficiency, malignancies, and HIV/AIDS^8,9^.

In recent years, due to the great progress in the detection and quantification of peptides in biological matrices, the information about peptide hormones has increased dramatically. Large amounts of data is scattered, and it is difficult to access it centrally. There are many databases related to peptide (such as DRAMP^10^, CancerPPD^11^, APD^12^, CAMP_R3_^13^ etc.). These databases are used to store data on antimicrobial and anticancer peptides, but few databases on peptide hormones. As far as we know, Hmrbase^14^ is a database specializing in hormones and receptors, but the data have not been updated since 2009. AHD 2.0^12^ is the Arabidopsis thaliana plant hormone database, which does not contain peptide hormones data. In order to devote ourselves to the development of peptide hormones, we have built HORDB. As a comprehensive database of peptide hormones, HORDB not only collects the latest reported peptide hormones, but also comes from a wider range of sources. Such as from plants, animals, especially humans. Each entry consists of six categories: basic information, sequence, structure, activity, physics and documents. The database also includes information about peptide hormones in the market. We believe that with the development of artificial intelligence in peptide design, the data sets provided by HORDB will make it easier for researchers to develop prediction models of peptide hormones and accelerate the discovery of peptide hormones.

HORDB is an open user-friendly database. Includes two main data sets, peptide hormones and peptide hormones drug data set. Users can obtain peptide or drug information through simple, advanced search or browsing. The collection of sequence analysis tools facilitates the comparison of peptides with known sequences. HORDB covers a wide range, including plants, animals and humans, with rich information types, which is helpful for the development and utilization of peptide hormones. We have chosen the CC0 1.0 license and shared the data of HORDB with researchers. HORDB is available freely for public from http://hordb.cpu-bioinfor.org.

## Methods

### Data collection and compilation

The peptide hormones of HORDB are collected from PubMed, Google Scholar, UniProt^15^ and PDB^16^ by using keywords such as “hormone” and “peptide hormones”. These matches are registered in the database if the following conditions are met: (1) the known amino acid sequence of the peptide; (2) the length is less than 100 amino acids; (3) mature peptide sequence without the precursor and signal regions; (4) a function annotation as hormone or phytohormone from literature or database such as UniProt or PDB. The information of peptide hormones drugs extracted from the literature and DrugBank^17^ in accordance with the above requirements. Except for the calculation of physical and chemical parameters using SciDBMaker^18^, the rest of the information (like activity, 3-dimensional structure, sequence, etc.) is collected from literature and databases such as PDB^16^, Uni-Prot^15^, AlphaFold DB^19^, etc.

### Database construction

**Fig. 1:**
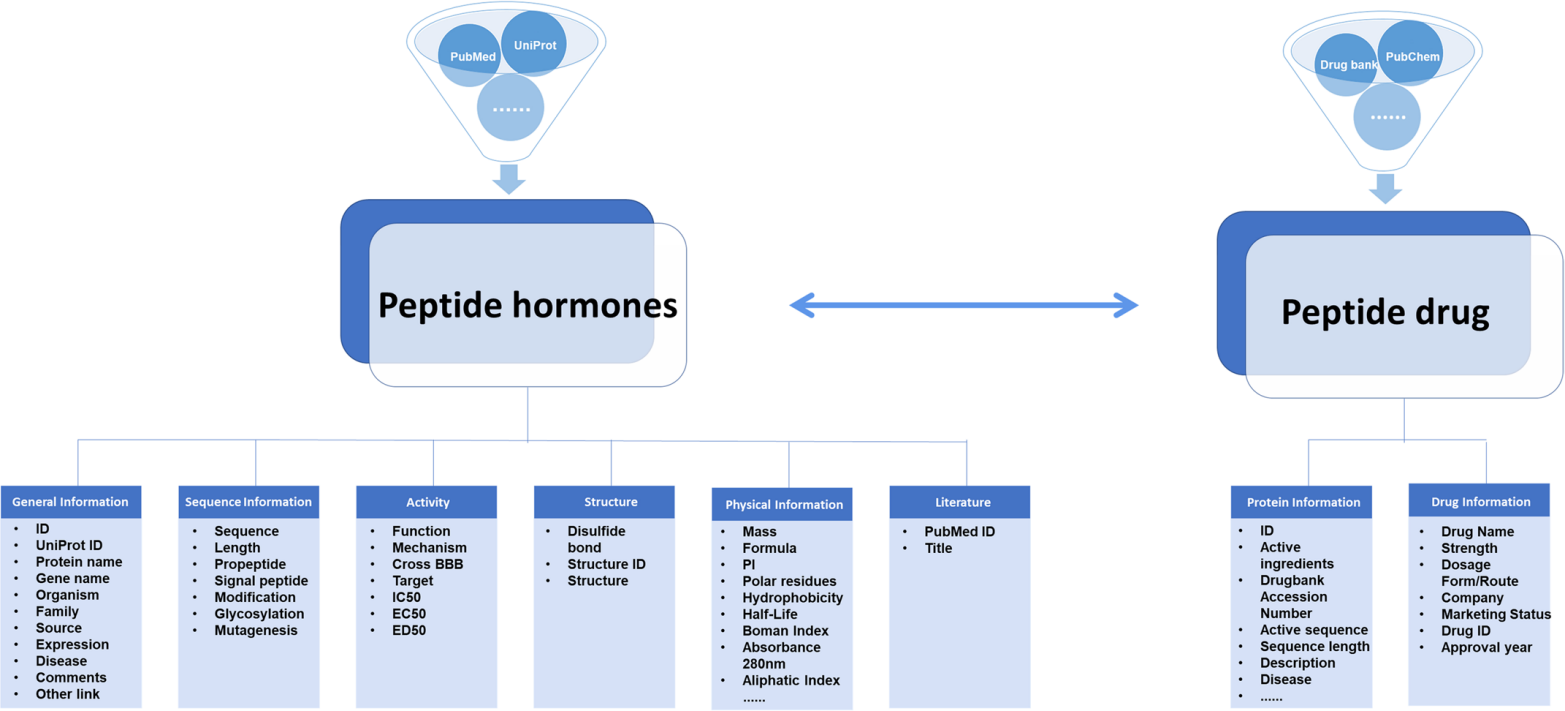
Architecture of the datasets in HORDB.

The HORDB database is established on the standard platform of Linux-Apache-MySQL-PHP (LAMP) with Linux as the operating system, Apache(version 2.2.22) as the server, MySQL server (version 5.5.29) as data management. HTML, PHP and JavaScript are applied to develop the front-end web interface. The architecture of HORDB database is given in Fig. 1.

## Data Records

### Peptide information

**Fig. 2:**
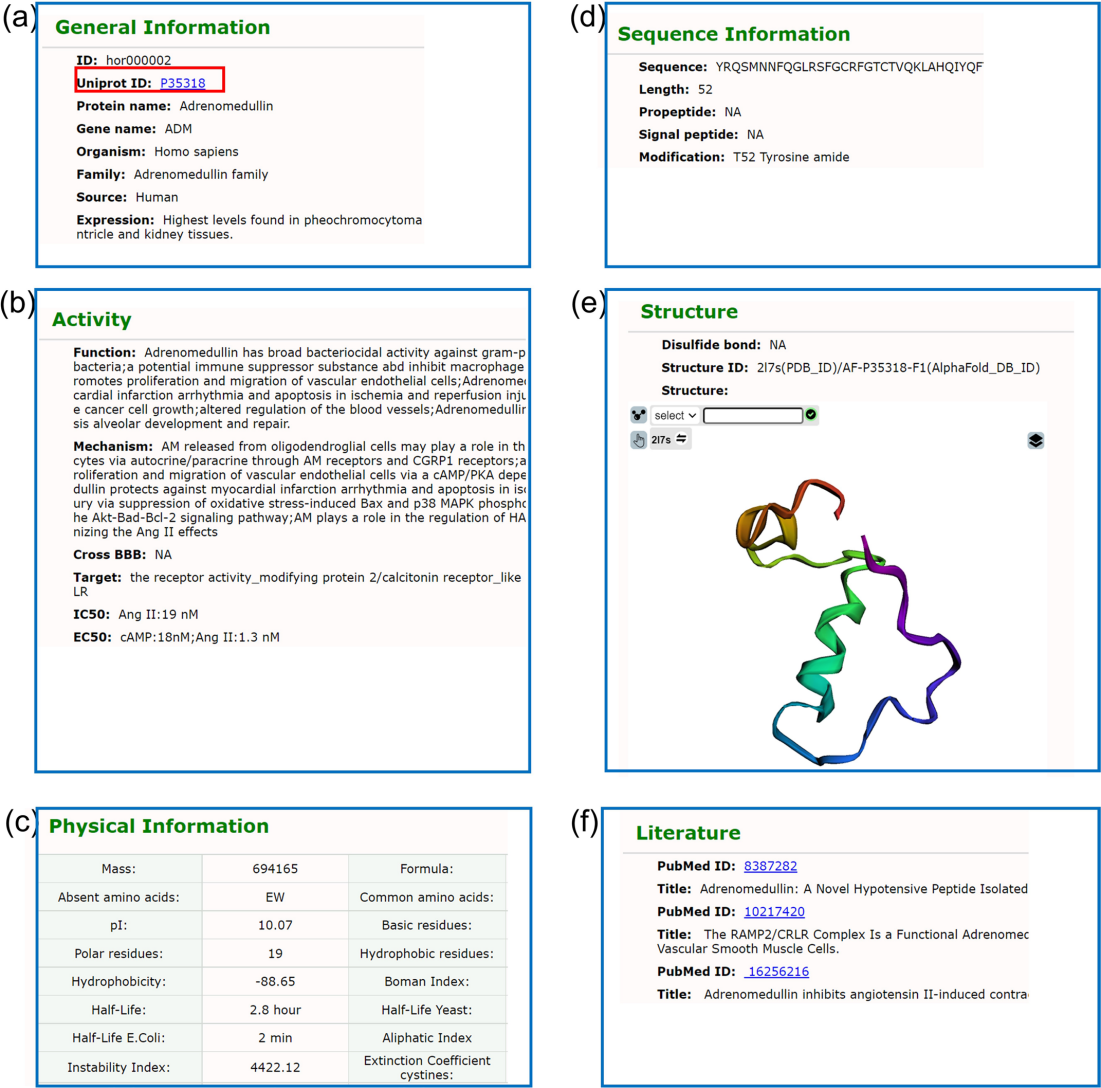
Peptide information section screenshot. (**a**) General Information; (**b**) Sequence Information; (**c**) Physical Information; (**d**) Sequence Information; (**e**) Structure; (**f**) Literature.

information about peptide hormones is obtained manually from different sources. Each entry contains six parts: general information (ID, UniProt ID, protein name, gene name, source, family, disease, comments, external link), sequence information (sequence, length, pro-peptide, signal peptide, modification), activity (function, mechanism, target, IC_50_/EC_50_/ED_50_, cross BBB), Structure, physical information, literature (PubMed ID, title). In order to make the page more user-friendly, users can jump to related notes by clicking on the left navigation bar. Among them, the function information is the physiological function of the peptide hormones in the organism. The information in the structure information includes the 3-dimensional structure; The disulfide bond position point information indicates the existence of a disulfide bond at two amino acid positions by marking them. As shown in Fig. 2.

### Drug information

**Fig. 3:**
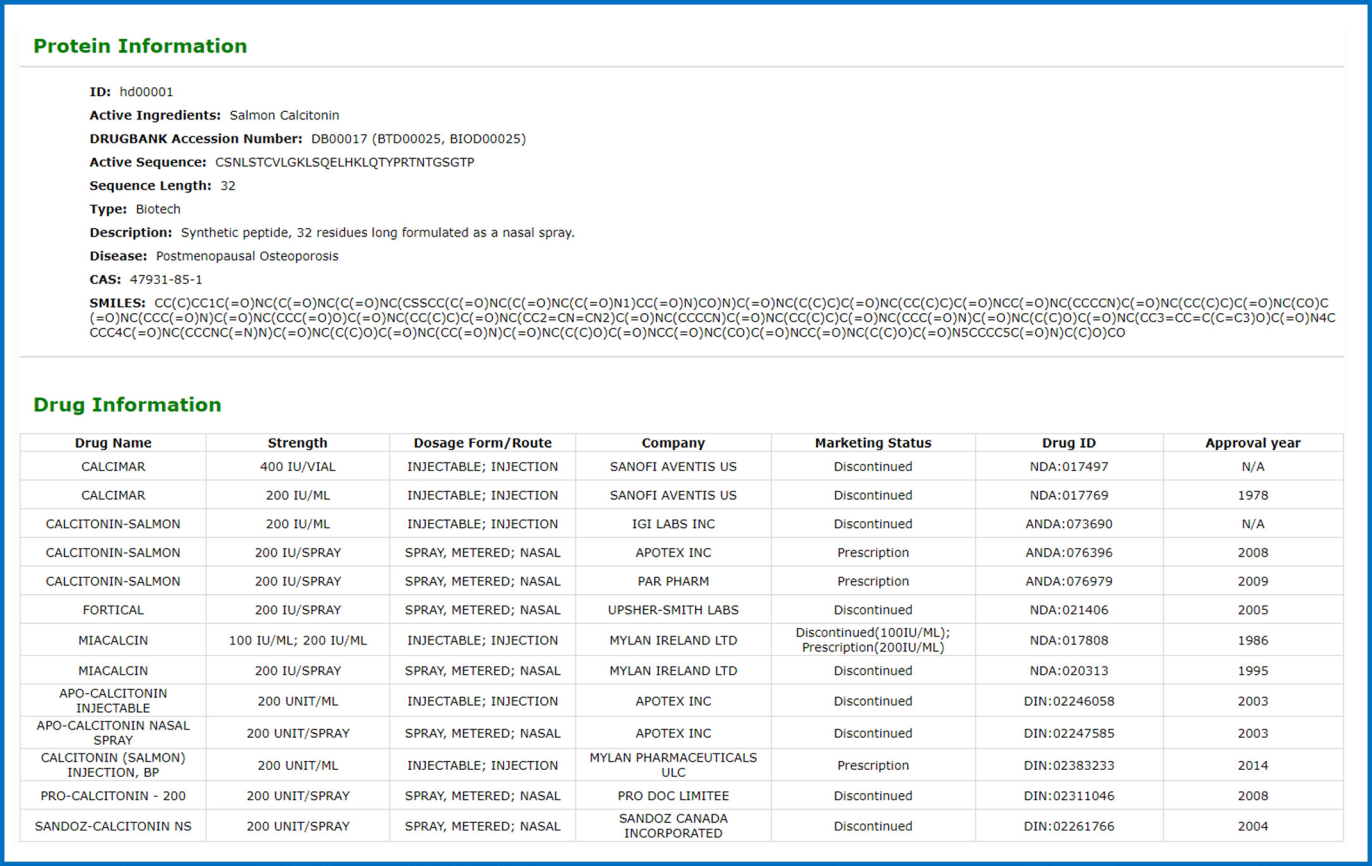
Screenshot of drug information details.

**Fig. 4:**
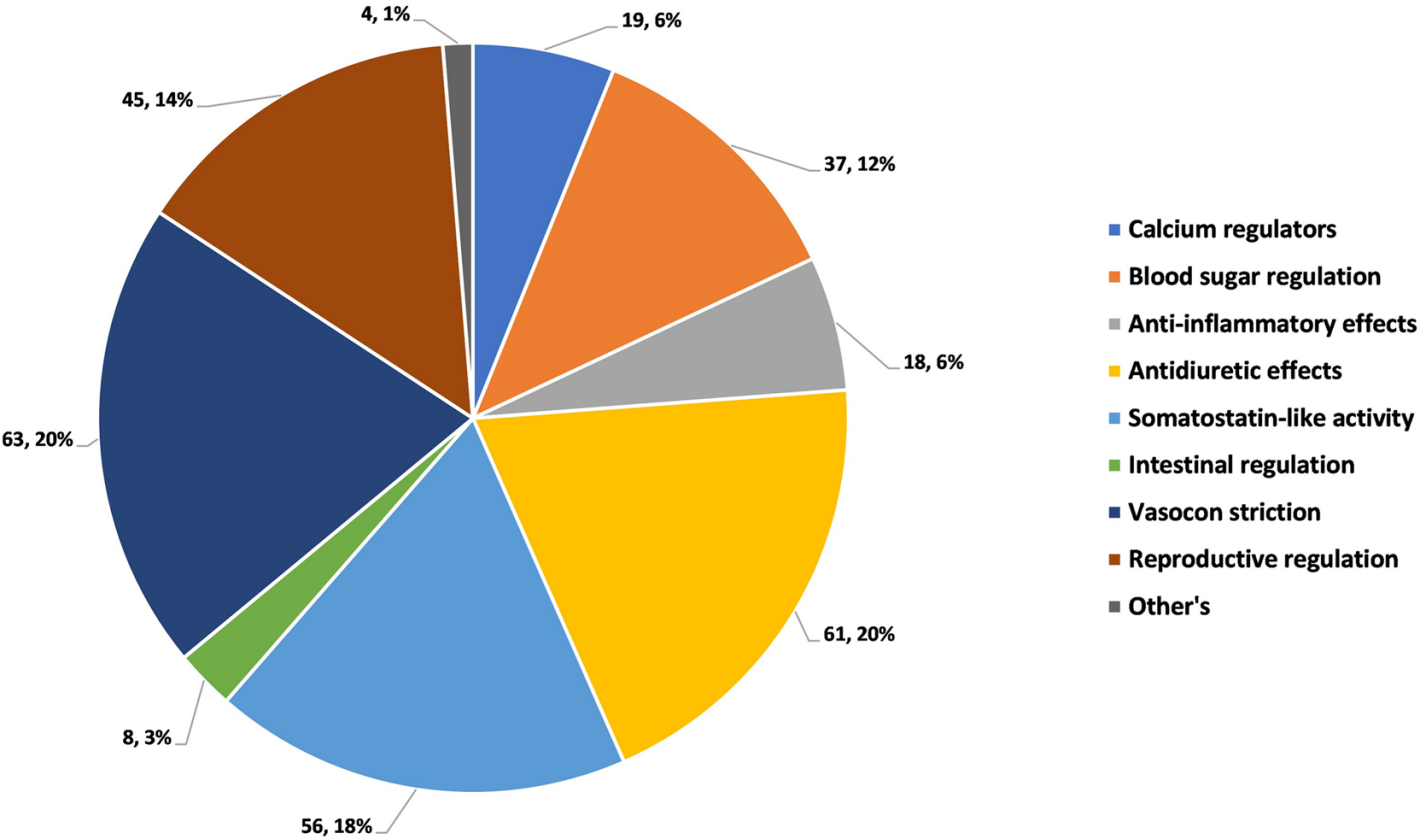
Functional distribution of peptide hormones in HORDB.

The drug information includes the following fields: ID, active ingredients, DrugBank Accession Number, Active Sequence, Sequence Length, Type, Description, Disease, CAS, Drug. The drug field embodies the drugs that the protein has been on the market: drug name, strength dosage form/route, company marketing status, Drug ID, approval year. Among them, the types of drugs are divided into small molecule drugs and biosynthetic drugs. The peptide hormones drug information collected in this database mainly comes from the DrugBank database and literature reports. The drug information is shown in Fig. 3. According to their functions, these drugs are manually sorted and analyzed manually. They can be divided into calcium regulators, blood sugar regulation, anti-inflammatory effects, antidiuretic effects, somatostatin-like activity, intestinal regulation, vasoconstriction, reproductive regulation and others, among others. The category includes reducing fat accumulation and dilating blood vessels. As shown in Fig. 4.

**Fig. 5:**
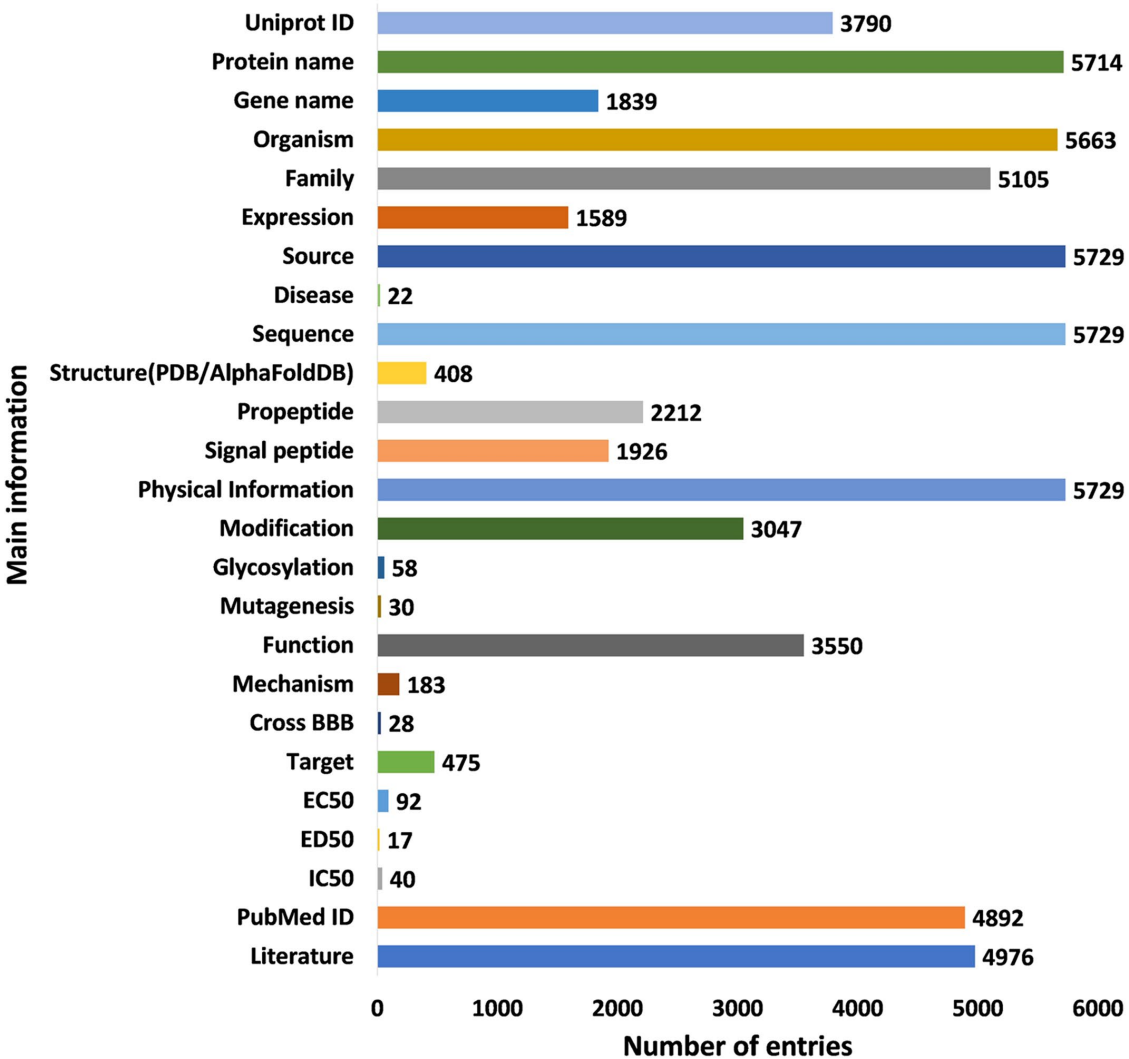
Number of entries corresponding to the main information.

According to statistics, the HORDB database has a total of 6024 entries, including 5729 peptide hormones, 40 peptide drugs and and 255 marketed pharmaceutical preparations information. There were 408 three-dimensional structure files of peptide hormones from the PDB^16^ (54 structures) and the AlphaFold DB^19^ (354 files), respectively. The statistical results of the main information entries are shown in the Fig. 5.

All of our data sets were stored as tables, compressed packages, and text in figshare^20^. “Peptide structure.zip” and “Peptide sequence.fasta.txt” for peptide structure (.pdb) and sequence information file (.fasta), respectively. Other excel files are used to store various kinds of information of peptide hormones, such as “Peptide hormones infomation.xlsx” used to store information of peptide hormones such as name, organization, source, activity, expression, structure, and so on; “Peptide Physical Information.xlsx” used to store information of physicochemical properties of peptide hormones. “Peptide Hormones Drugs Information.xlsx” and “Marketed Pharmaceuticals preparations infomation.xlsx” are used to store information on peptide drugs and their preparations. At the same time can also through our online database website browsing, use, download data, we will regularly update the data. The website is http://hordb.cpu-bioinfor.org.

## Technical Validation

### Data validation

We manually proofread and validate the main data of HORDB at the back end of the website. For peptide hormones data, we re-validated the number of primary information entries based on the MySQL metadata information. Check the accuracy of information by random sampling. For the metadata of MySQL database, in order to facilitate researchers and subsequent data sorting and updating, it was divided into four data tables, namely, peptide information master data (hor_gen), active pep (Active_PEP), peptide physical data (hor_phs) and market drug preparation data. All datasets are available on the download page.

### Data update

In addition to our regular updates, we also set up a submission page to encourage researchers to participate in HORDB data updates as contributors. Researchers can also give us any advice through the contact information on the homepage. With the deployment of the HORDB, we plan to update the data about every three months. In addition, when major changes that require version control occur, the production version of the database will be updated regularly.

## Usage Notes

**Fig. 6:**
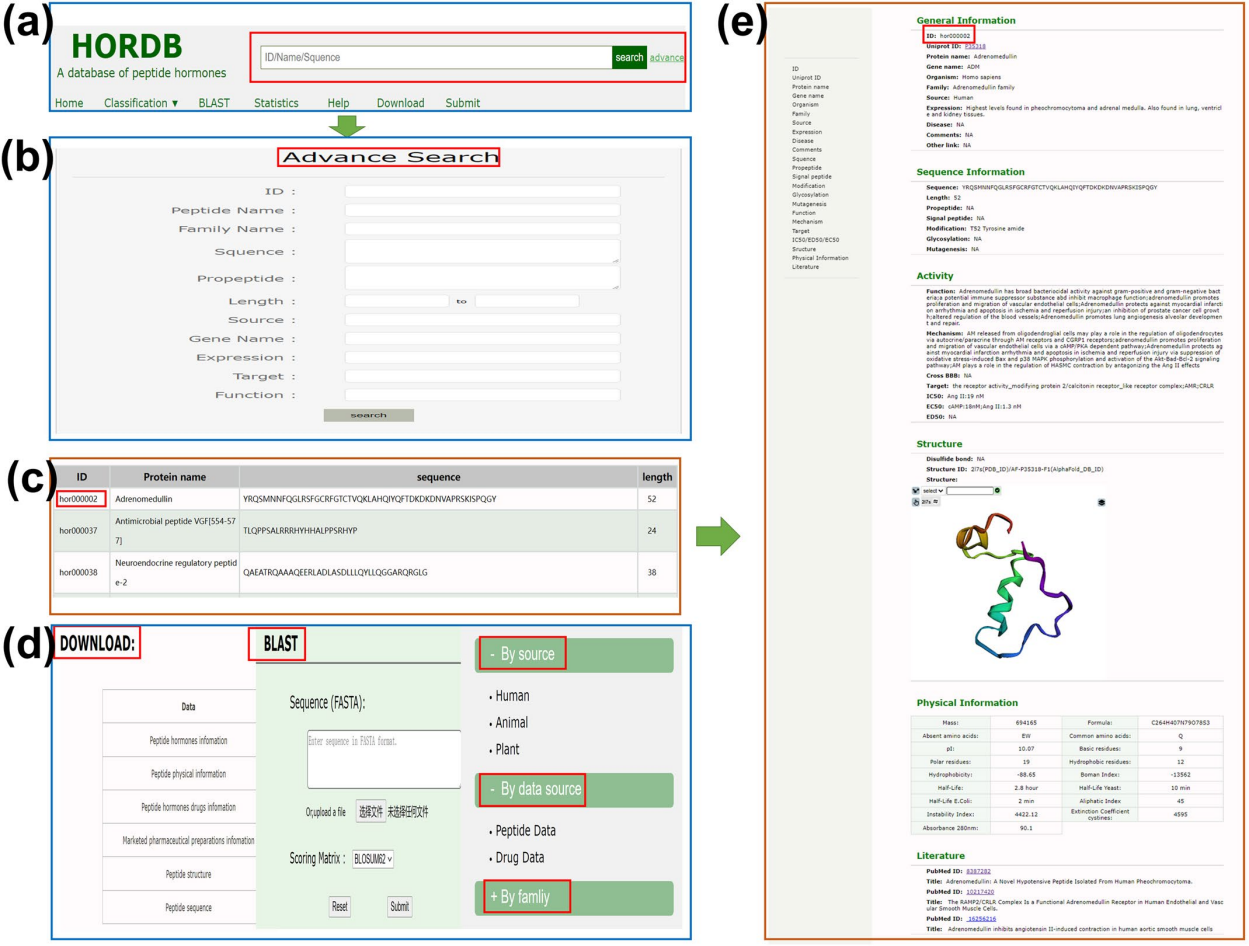
Screenshots of HOROB web interfaces. (**a**) Basic navigation bar of HOROB homepage; (**b**) Shows the advanced search page.This interface allows users to query database by a combination of various conditions; (**c**) Display the result of query or browsing; (**d**) The download, browse list and BLAST^21^ analysis interface is displayed; (**e**) Shows the detailed information page.

The homepage of HOROB contains the following interfaces: search, browse, tools, statistics, downloads and links. A brief description of the interface is given below, and a screenshot is given in Fig. 6.

### Data retrieval or search tools

The database integrates simple search and advanced search to meet the different needs of users. Entering ID, name or sequence in the simple search box at the top of the page could retrieve the database. On the advanced search page, users combine keywords like ID, peptide name, family name, sequence, pro-peptide, length, source, target and other keywords to submit query content according to their requirements. The main web page of HORDB contains the following interfaces: Search, Browse, Tools, Statistics, Download and Links.

### Browse

To facilitate browsing, the database provides three cross-linked browse tables. (1) on the basis of biological sources, it is divided into plants, animals and humans; (2) on the basis of the data source, it is divided into peptide data and drug data. (3) on the basis of family classification, there are a total of 80 different family categories.

### Sequence alignment

The HORDB database furnishes the blast tool. Users submit their peptide sequences in FASTA format. The server performs a BLAST^21^ search on the users’ query sequence for the amino acid sequence of all peptide in the database.

### Download

We are willing to share our data with users, so the website is designed with a download interface. The downloadable interface is divided into four parts: general information, physical information, peptide drug information, peptides sequence, peptide structure and listed drug information.

### HORDB data statistics

**Fig. 7:**
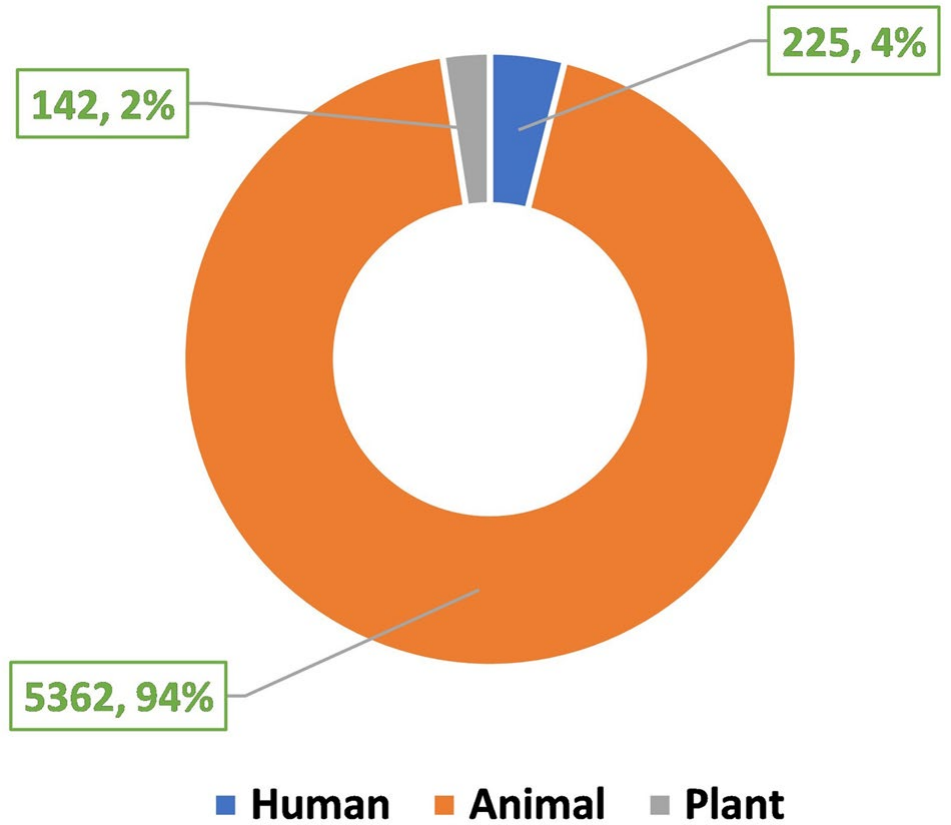
Organsim distribution of peptide hormones.

**Fig. 8:**
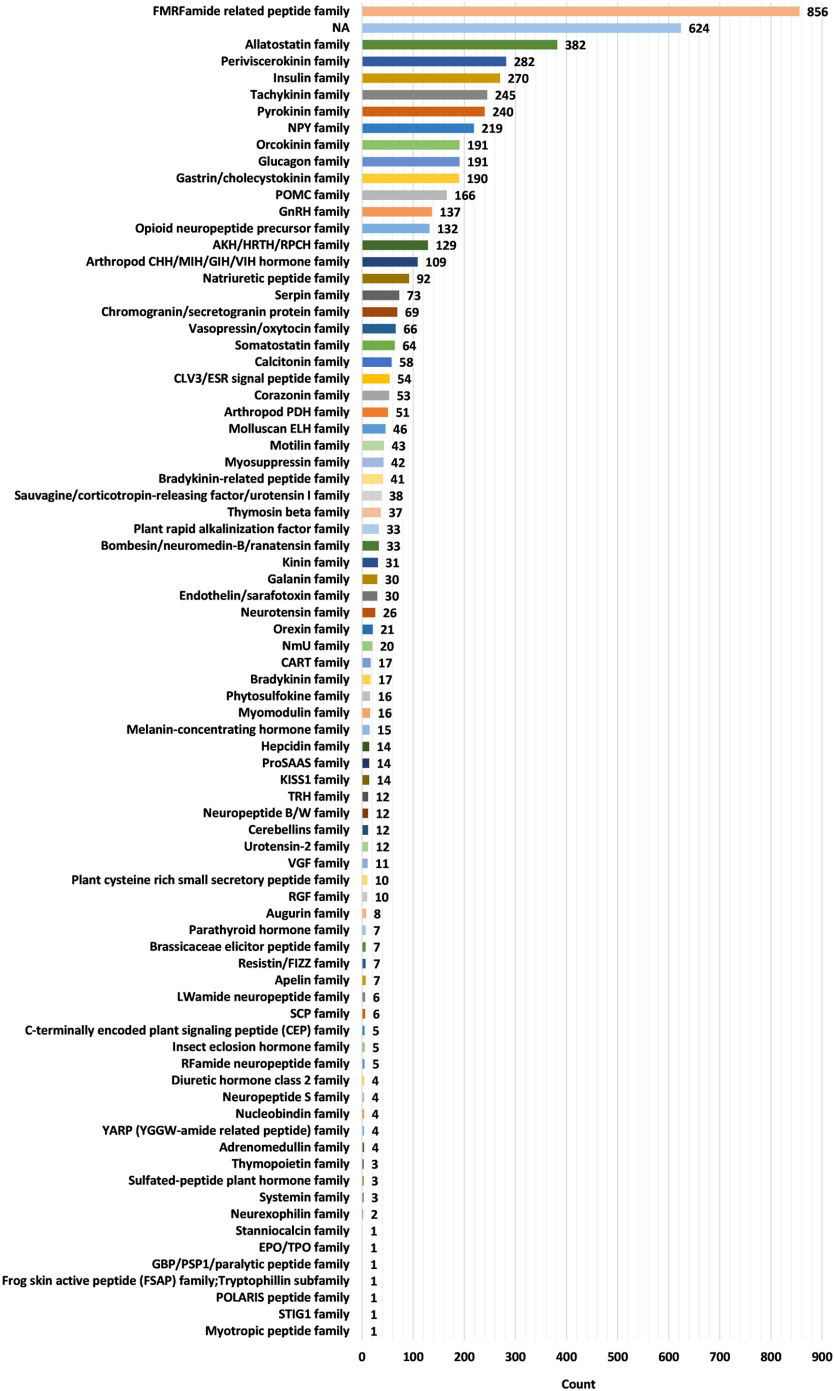
Family Classification of peptide hormones.

**Fig. 9:**
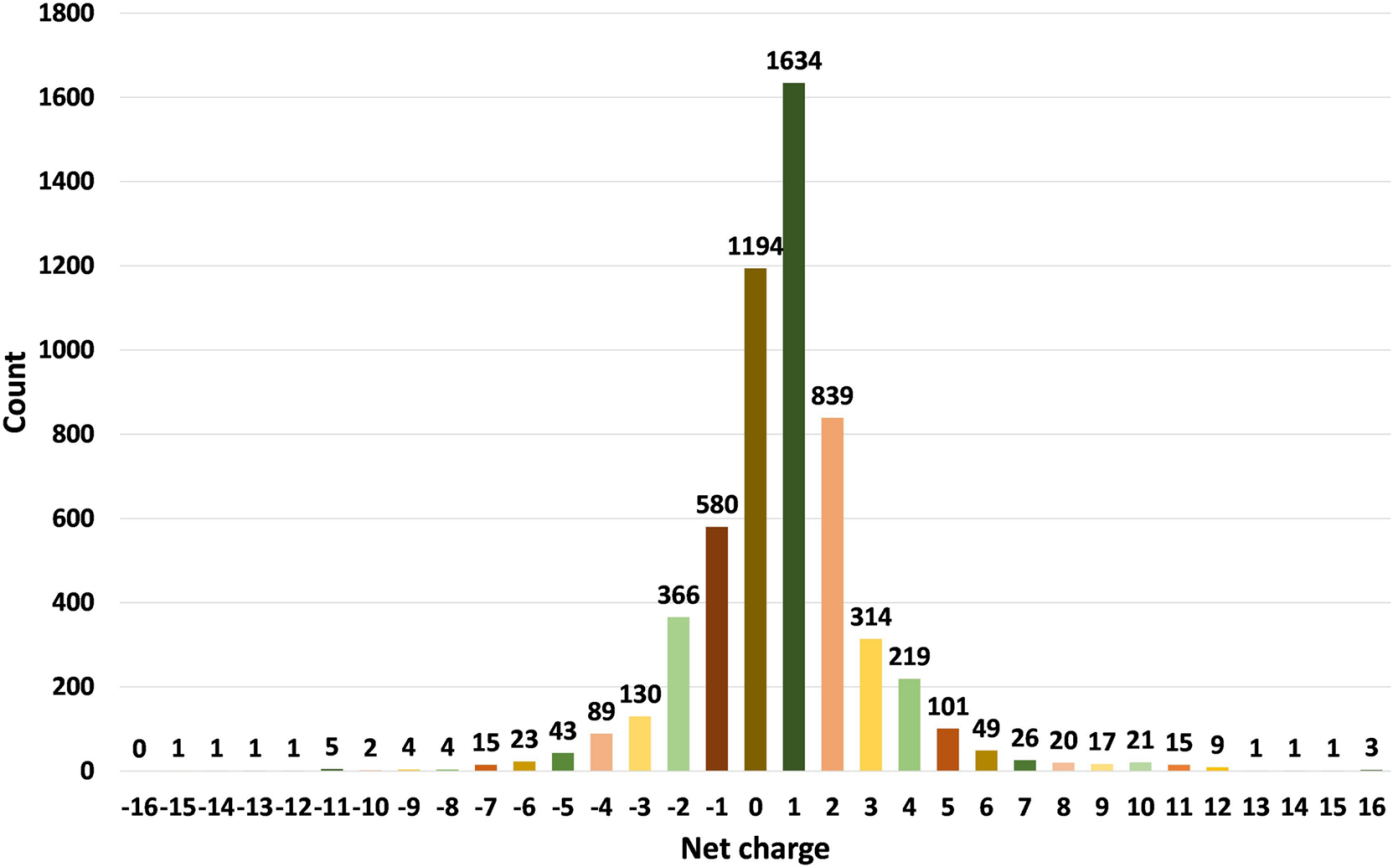
Net charge distribution of peptide hormones.

**Fig. 10:**
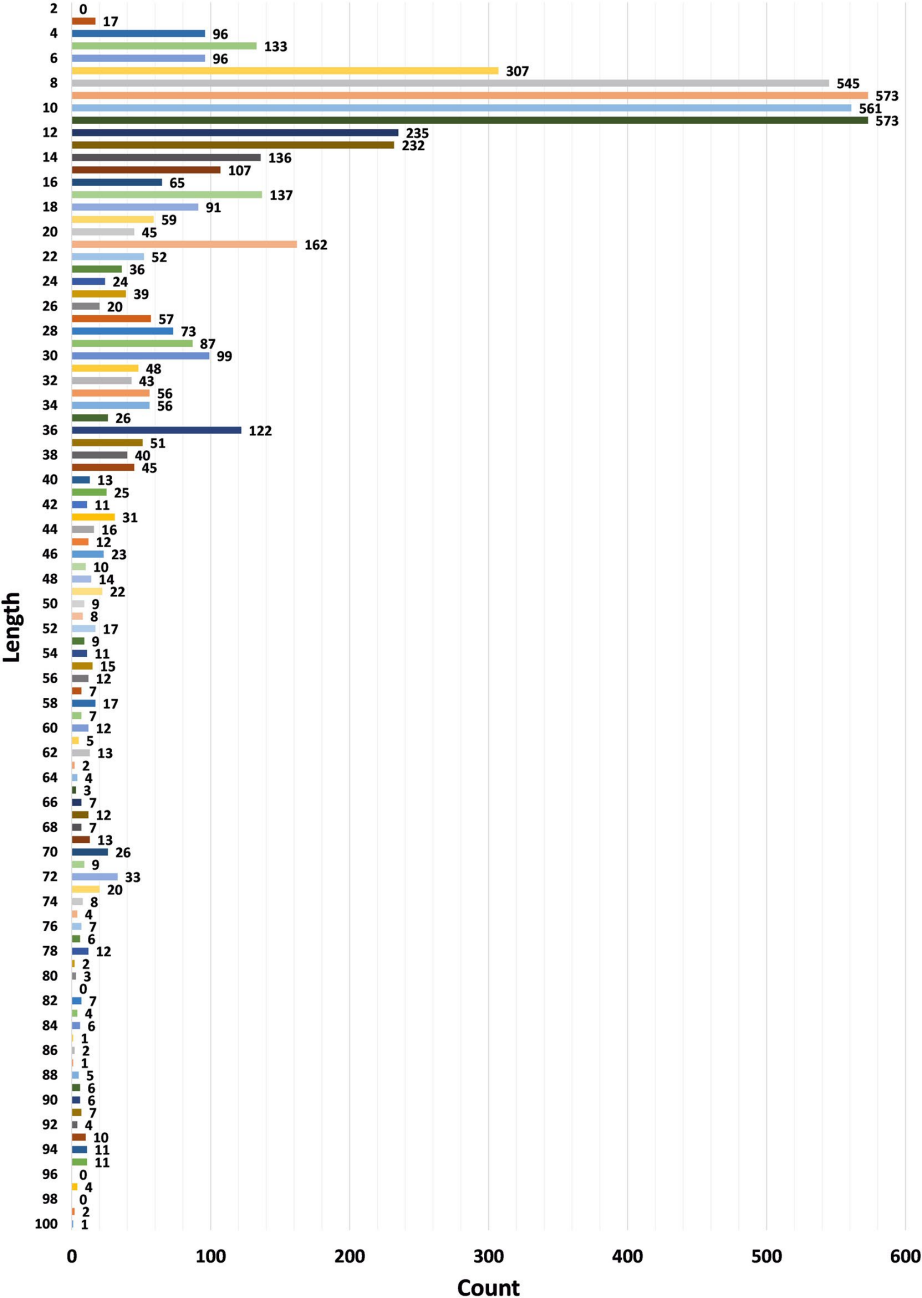
Length distribution of peptide hormones.

**Fig. 11:**
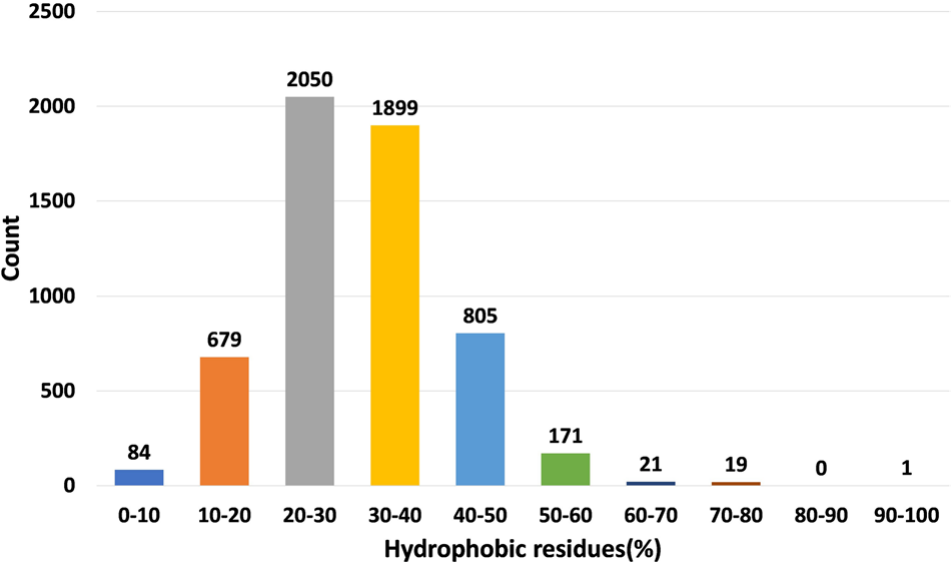
Hydrophobic residue distribution of peptide hormones.

**Fig. 12:**
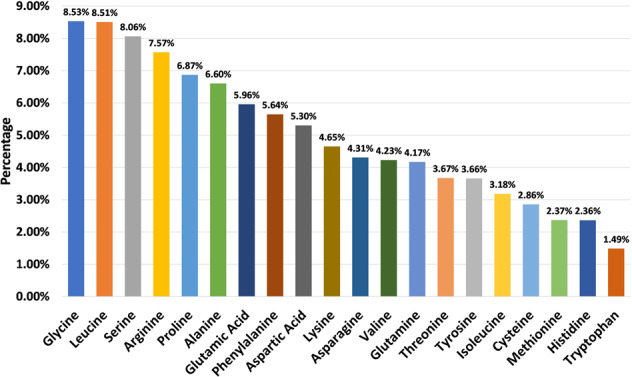
Amino acid distribution of peptide hormones.

The current version of the HORDB database contains 6,024 entries, including 5,729 peptide hormones, 40 peptide drugs, and 255 pharmaceutical preparations on the market. 408 peptide hormones had available three-dimensional structures; They are collected from the PDB^16^ and AlphaFoldDB^19^. Among them, 28 peptide hormones could cross the blood-brain barrier; 22 peptide hormones with clear disease information. In order to improve the understanding of the characteristics of peptide hormones and serve as the basis for the design of peptide hormones, the peptide sequences in the database were analyzed and counted. In the database, most peptide were derived from animals, as shown in Fig. 7. According to the protein classification criteria of UniProt^15^, there are 80 families in the HORDB. The most abundant (856 in total) were the FMRFamide related peptide families, and the distribution of peptide hormones families is shown in Fig. 8. The peptide hormones shown in Fig. 9 is mostly positively char. Among the peptide with known sequences, most peptide are about 10 amino acids in length (as shown in Fig. 10). The results showed that short peptide formed peptide hormones more easily than long peptide. As shown in Fig. 11, the proportion of hydrophobic residues in most peptide is 20–40%, indicating that most peptide hormones in HORDB are less hydrophobic. The distribution of amino acids is shown in Fig. 12. Arginine, glycine, leucine and serine are the main residues of natural peptide hormones. These findings may contribute to the development of peptide hormones models or the design of novel peptide hormones with higher activity.

## Data Availability

The source code for the HORDB database website has been uploaded to GitHub: https://github.com/CPU-HORDB/HORDB.
